# An ancestral host defence peptide within human β-defensin 3 recapitulates the antibacterial and antiviral activity of the full-length molecule

**DOI:** 10.1038/srep18450

**Published:** 2015-12-21

**Authors:** Ersilia Nigro, Irene Colavita, Daniela Sarnataro, Olga Scudiero, Gerardo Zambrano, Vincenzo Granata, Aurora Daniele, Alfonso Carotenuto, Stefania Galdiero, Veronica Folliero, Massimiliano Galdiero, Richard A. Urbanowicz, Jonathan K. Ball, Francesco Salvatore, Antonello Pessi

**Affiliations:** 1 CEINGE-Biotecnologie Avanzate, Via Gaetano Salvatore 486, 80145 Napoli, Italy; 2 Department of Molecular Medicine and Medical Biotechnology, University of Naples Federico II, Via Sergio Pansini 5, 80131 Napoli, Italy; 3 Department of Environmental, Biological and Pharmaceutical Sciences and Technologies, Second University of Naples, Via Vivaldi 43, 81100 Caserta, Italy; 4 Department of Pharmacy, University of Naples Federico II, Via Domenico Montesano 49, 80131 Napoli, Italy; 5 Institute of Biostructures and Bioimages, CNR, Naples, Italy; 6 Department of Experimental Medicine, Second University of Naples, Via Costantinopoli, 16, 80138 Napoli, Italy; 7 The School of Life Sciences and the Nottingham Digestive Diseases Centre Biomedical Research Unit, The University of Nottingham, Queen’s Medical Centre, Nottingham NG7 2UH, United Kingdom; 8 IRCCS-SDN Foundation, Via Emanuele Gianturco 113, 80142 Napoli, Italy; 9 PeptiPharma, Viale Città D’Europa 679, 00144 Roma, Italy

## Abstract

Host defence peptides (HDPs) are critical components of innate immunity. Despite their diversity, they share common features including a structural signature, designated “γ-core motif”. We reasoned that for each HDPs evolved from an ancestral γ-core, the latter should be the evolutionary starting point of the molecule, i.e. it should represent a structural scaffold for the modular construction of the full-length molecule, and possess biological properties. We explored the γ-core of human β-defensin 3 (HBD3) and found that it: (a) is the folding nucleus of HBD3; (b) folds rapidly and is stable in human serum; (c) displays antibacterial activity; (d) binds to CD98, which mediates HBD3 internalization in eukaryotic cells; (e) exerts antiviral activity against human immunodeficiency virus and herpes simplex virus; and (f) is not toxic to human cells. These results demonstrate that the γ-core within HBD3 is the ancestral core of the full-length molecule and is a viable HDP *per se,* since it is endowed with the most important biological features of HBD3. Notably, the small, stable scaffold of the HBD3 γ-core can be exploited to design disease-specific antimicrobial agents.

Host defence peptides (HDPs) are a critical component of innate immunity, and represent a first line of defence against infection by a broad spectrum of pathogens. HDP expression is found in the host tissues most exposed to microorganisms (skin and internal epithelia of e.g. the respiratory and gastrointestinal tracts) and in the cells of the immune system (macrophages, lymphocytes, platelets etc.)^1^. Since a number of pathogens that are refractory to conventional antibiotics are sensitive to HDPs, there is considerable interest in the development of these peptides as therapeutics^2^. Moreover, it is becoming increasingly clear that these multifunctional peptides exert other functions besides antimicrobial action, for example, they are involved in the immune surveillance against cancer^3^. Accordingly, almost 1,000 different HDPs have been identified^4^. Despite this diversity, all HDPs share the following features: a small size (<10 kDa), a positive charge at neutral pH, and an amphipathic structure. This secondary structure drives the interaction of HDP with lipid bilayers and, critically, it enables selectivity between the bacterial membranes and the cholesterol-rich eukaryotic cell membranes. The mechanistic aspects of these molecules are important aspects for their function in biological systems^5^,^6^,^7^,^8^.

Yount & Yeaman identified another common structural signature in the broad sub-family of HDPs stabilized by cysteine bridges, which they named the “γ-core motif”^2^,^9^,^10^. The presence of the γ-core not only in antimicrobial peptides, but also in peptide toxins and venoms, in microbicidal chemokines (kinocidins), and in plant thionins^9^,^11^,^12^ supports the hypothesis that it may represent an archetypal membrane-binding domain present in a common ancestor of this family of cysteine-stabilized HDPs^9^,^11^,^12^. Preservation of the γ-core motif structure despite a high level of sequence variability may have enabled the evolution of a broad range of HDPs with additional and/or specialized activities^11^. We reasoned that if such an evolutionary mechanism has been at play in the generation of current HDPs, one would expect the γ-core motif *within* a given HDP to be the evolutionary starting point of the full-length molecule, and thus itself be a primordial HDP. To test this hypothesis, we explored the γ-core motif of human β-defensin 3 (HBD3).

Human β-defensins (HBDs) are defined by a conserved triple-disulfide scaffold with Cys^1^–Cys^5^, Cys^2^–Cys^4^ and Cys^3^–Cys^6^ connectivities, with otherwise little sequence conservation among species. They are produced mainly in epithelial tissues including skin, lung^13^ and oral^14^ epithelium, and provide a multimodal first line of defence against invading pathogens^15^,^16^,^17^. Besides exerting an antimicrobial effect, these multifunctional peptides are also involved in fertility, development, wound healing, and cancer^18^. Among inducible HBDs, HBD3 is particularly attractive because it has a low minimum inhibitory concentration for antibacterial activity and it maintains potency in the presence of high salt concentrations, whereas the other HBDs are inactivated in these conditions^16^.

We previously demonstrated that chimeric peptides of HBD1 and HBD3 have both high potency and salt resistance^19^,^20^. More recently we identified an indirect mechanism of antibacterial action of HBD3 at epithelial surfaces based on competitive binding to CD98, a cell surface receptor utilized by intestinal bacteria during invasion of colonic tissue. Binding to CD98 leads to ready cell internalization of HBD3, and to downregulation of the protein expression^21^. In addition to antibacterial activity, HBD3 also exerts antiviral activity through a variety of mechanisms^22^,^23^,^24^,^25^. In particular, it is active against such enveloped viruses as human immunodeficiency virus (HIV), herpes simplex virus (HSV)^26^,^19^, influenza A virus^24^, and vaccinia virus^27^. Interestingly, CD98 plays a regulatory role in membrane fusion mediated by the enveloped viruses Newcastle disease virus^28^, human parainfluenza virus type 2^29^, HIV^30^,^31^, and HSV^32^, suggesting that binding to and downregulation of CD98 by HBD3 may be an antiviral, in addition to an antibacterial, mechanism. Finally, HBD3 is a high-affinity ligand for melanocortin receptors^33^,^34^, although the biological significance of this process is unclear. This pleiotropy of biological activities suggests that HBD3 may be an excellent example of the evolutionary strategy based on the γ-core^11^ whereby the latter is the structural scaffold endowed with basal antimicrobial activity onto which additional α-helical and/or β-sheet domains are added in a modular fashion to improve potency and/or to exert novel functions. Moreover, the structure of HBD3 and its variants have been extensively studied^19^,^20^,^21^,^22^,^23^,^24^,^25^,^26^,^27^,^28^,^29^,^30^,^31^,^32^,^33^,^34^,^35^,^36^,^37^.

In this article, we report that a peptide corresponding to the γ-core of HBD3 is endowed with direct and indirect antibacterial and antiviral activity, and has a low cytotoxicity − all features typical of the parent β-defensin. We also report that the HBD3 γ-core peptide folds rapidly and is highly stable in human serum.

## Results

### Role of the γ-core in folding of full-length HBD3

**Figure 1:**
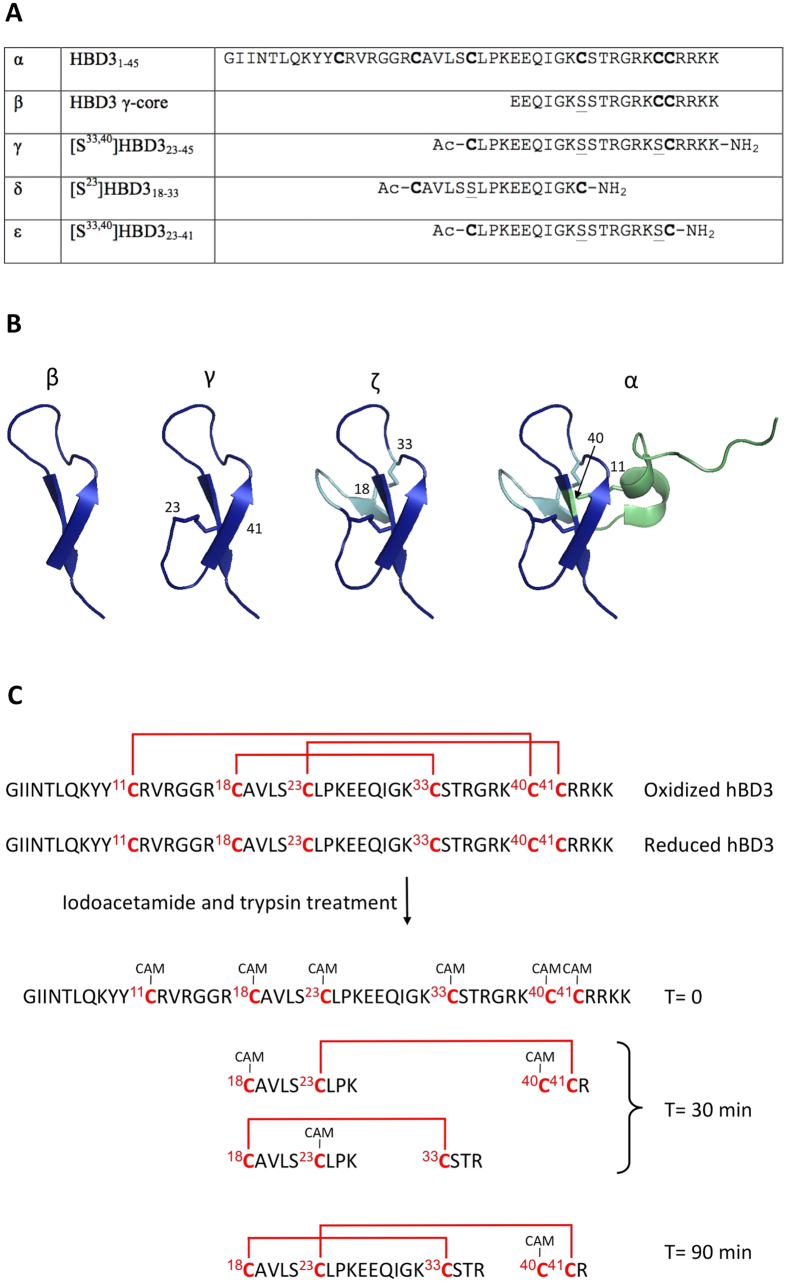
Modular structure of HBD3 and γ-core peptides. (**A**) Sequences of the peptides used in this study. In the peptides, Cys^23^, Cys ^33^ and Cys^40^ (HBD3 numbering) are mutated to Ser. (**B**) peptide β: theγ-core motif of HBD3 as identified bioinformatically by Yount & Yeoman (Yount and Yeaman, 2004); peptide γ: theβ-hairpin enclosed by the disulfide Cys^23^-Cys^41^ which encompasses the γ-core; peptide φ: theCys^23^-Cys^41^ β-harpin containing the γ-core motif (blue) linked to the third strand of the β-sheet (cyan) by the Cys^18^-Cys^33^ disulfide; peptide α: the β-sheet containing the γ-core motif is linked to the α-helical domain (green) through the Cys^11^-Cys^40^ disulfide to complete the full-length peptide. The figure was built with Pymol using for HBD3 the PDB code 1KJ6 (Schibli, *et al.*, 2002). (**C**) Time-course of the oxidative folding of 0.5 mg/mL HBD3 in Tris HCl, pH 7.5. Aliquots of the folding mixture at various time points were treated with iodoacetamide, and excess reagent removed by HPLC. Following trypsin treatment, fragments were identified by MS. Shown is the structure of the fragments found at the indicated times, which define the sequence of disulfide bond formation during refolding. See also Figures S1, S2, Tables S1–S4.

The γ-core motif of HBD3 as identified by Yount & Yeaman^9^ is shown in Fig. 1B. It is part of a β-hairpin closed by the native disulfide Cys^23^-Cys^41^. This β-hairpin takes a central position in the molecule, being bridged on one side to a third strand of an antiparallel β-sheet, and on the other side to the α-helical domain. Cys^33^ and Cys^40^ within the β-hairpin link to Cys^18^ in the third strand of the β-sheet and to Cys^11^ in the α-helical domain, respectively. We hypothesized that the β-hairpin including the γ-core may represent the evolutionary starting point and the minimal active substructure of HBD3 and if so, it should represent the folding nucleus of full-length HBD3. To address this hypothesis, we studied the oxidative folding of fully reduced HBD3 in benign medium at physiologic pH (Tris HCl pH 7.5).

At various time points, aliquots were removed and treated with iodoacetamide, to alkylate any cysteines not yet engaged in disulfide bond formation; the fragments formed upon trypsin treatment were then identified by mass spectrometry (Fig. 1C; see also Figure S1 and Table S1). A control sample at time zero confirmed that all six cysteines were efficiently alkylated in the absence of disulfides (data not shown).

After 30 min, we identified trypsin fragments which unambiguously showed the simultaneous formation of either one of two disulfides: Cys^23^-Cys^41^ and Cys^18^-Cys^33^. After a further 60 min, both disulfides were present in the same molecule, while Cys^11^ and Cys^40^, that bridge the β-sheet to the α-helix, were alkylated. Formation of the Cys^11^-Cys^40^ disulfide was the last event in the oxidative folding: after 180 minutes, the molecule with three disulfide bridges started to appear, while the 2-disulfide peptide was still present (Figure S1, Table S1). These data show that formation of the γ-core β-hairpin closed by the Cys^23^-Cys^41^ disulfide occurs, as expected, at the beginning of the folding pathway of HBD3, together with the β-hairpin closed by the Cys^18^-Cys^33^ disulfide. In the full-length molecule, formation of the three-stranded β-sheet likely aligns both Cys^23^ with Cys^41^ and Cys^18^ with Cys^33^, ensuring rapid disulfide bond formation. Whichever disulfide is formed first, it is rapidly followed by formation of the other one to yield the 2-disulfide peptide.

### β-hairpin peptides within HBD3

To identify which of the two initially formed β-hairpins actually nucleates the β-sheet, we synthesized and studied the corresponding peptides, i.e. HBD3_23–45_ and HBD3_18–33_. Within each peptide, the cysteines that in HBD3 project towards the additional structural elements, Cys^23^ in HBD3_18–33_ and Cys^33,40^ in HBD3_23–45,_ were mutated to serine. Peptides γ and δ in Fig. 1A correspond to [S^33,40^]HBD3_23–45_, i.e. to the γ-core β-hairpin, and the alternative β-hairpin [S^23^]HBD3_18–33_. The peptides were prepared with N-terminal acetyl and C-terminal carboxyamide, to maintain in the isolated peptide the net charge that the sequence has within HBD3.

### The γ-core disulfide forms rapidly in buffer at physiologic pH and in human serum

When formation of the intramolecular disulfide in peptides γ and δ was monitored in the same conditions used for full-length HBD3, we found that it formed rapidly in peptide γ, but very slowly in peptide δ: after 3 h, while peptide γ was completely oxidized, peptide δ was still mostly reduced (Figure S2). The strong tendency to form the Cys^23^-Cys^41^ disulfide was confirmed in human serum: when reduced peptide γ was incubated in human serum, oxidation to the disulfide-bridged form was even more rapid than in buffer, being complete within 60 min (Figure S3).

Taken together, the folding experiments strongly suggest that the γ-core β-hairpin represents the nucleating unit of HBD3, consistent with its putative role as the archetypal core of the molecule.

### The γ-core β-hairpin of HBD3 displays a flexible structure in solution

The structure of peptide γ in solution was analyzed in neutral aqueous buffer by nuclear magnetic resonance (NMR). The proton resonances were assigned according to established strategies^38^ using Double Quantum Filtered Correlated Spectroscopy (DQF-COSY) and Total Correlated Spectroscopy (TOCSY) spectra to recognize amino acid spin systems, and Nuclear Overhauser Enhancement Spectroscopy (NOESY) spectra to identify sequential connectivities. Low chemical shift dispersion resulted in extensive overlap of the resonances. As a result, exact assignment could not be achieved for many backbone and side chain proton resonances (Tables S2, S3). A number of observations suggest that the peptides exist in aqueous solution as a dynamic ensemble of multiple conformations. The backbone and side chain chemical shifts closely approach typical random coil values^39^(with a spread of Δδ < 0.2 ppm). Also, the Wishart-Sykes chemical shift index^40^ predicts no regular secondary structure on the basis of the observed α-proton chemical shifts. The observed ^3^J_Nα_ couplings range between 6.3–7.8 Hz again indicating conformational averaging^40^. The observed Nuclear Overhauser Effect (NOE) connectivities also confirm the disordered state of the peptides. Indeed, the consistent presence of both strong d_αN_(i,i + 1) and relatively strong d_NN_(i,i + 1) NOEs indicates that the peptides sample a broad range of conformations, despite the presence of the disulfide bond. In full-length HBD3, the γ-core of peptide γ is part of an antiparallel three-stranded β-sheet formed by residues 27–32 and 38–42 (Fig. 1B). None of the diagnostic NMR parameters (Hα downfield shifts, ^3^J_Nα_ > 8 Hz, d_αN_(i,j) and d_NN_(i,j) long range NOE’s) identified in a previous study for the β-sheet^41^ were present in our data set. For comparison, the α-proton chemical shift differences from the HBD3 resonances in reference^41^ are shown in Tables S2–S3. To investigate whether rigidification of the γ-core β-hairpin structure is induced by interaction of the peptide with a membrane, NMR spectra of peptide γ were also acquired in a membrane mimetic dodecyl-phosphocholine (DPC) micelle solution. Although a better signal dispersion was observed in the DPC solution compared to water, which allowed a higher number of resonance assignments, analysis of these spectra showed little variations in the NMR parameters (Table S4), indicating that the peptide is unstructured also in the presence of a membrane environment. It is noteworthy that solution NMR studies of large molecules and complexes are limited by the increased linewidths associated with slower tumbling. This is the case of lipid bilayers, which are not amenable to solution NMR studies. Now, micellar systems, such as DPC micelles, are commonly used as membrane mimetics in solution NMR studies. Protein interaction with near-native membranes like lipid bilayers can only be obtained using solid state NMR experiments like PISEMA. Several solid state NMR studies using aligned lipid bilayers to establish the membrane bound structure and orientation of the peptide have been reported^42^,^43^,^44^,^45^,^46^,^47^.

Overall, the NMR study shows that the γ-core β-hairpin has a flexible structure, which is rigidified only upon the addition of the third strand of the β-sheet.

### The γ-core β-hairpin of HBD3 is stable in human serum

HBD3 with its rigid, triple disulfide-stabilized structure, is highly stable in human serum^48^,^20^; the only site of vulnerability is the exposed R^42^-K^45^ C-terminal region^15^. We analyzed the γ-core peptide [S^33,40^]HBD3_23–45_ (peptide γ), which instead displays a flexible structure in solution, under the same conditions, and found very similar behaviour to the full-length molecule (Table S5). Peptide γ was completely stable up to 3 h of incubation. After this time, fragments began to appear resulting from C-terminal cleavage between Arg^20^–Arg^21^ and Lys^22^–Lys^23^ (Arg^42^–Arg^43^ and Lys^44^–Lys^45^ in HBD3 numbering), consistent with trypsin-like enzymatic activity, and between Cys^19^–Arg^20^ (Cys^41^–Arg^42^ in HBD3), likely due to carboxypeptidase activity following trypsin cleavage. No cleavage was observed within the sequence delimited by the disulfide bridge Cys^1^–Cys^19^, (Cys^23^–Cys^41^ in HBD3). At the end of the 24 h incubation period, the only peptides observed were intact [S^33,40^]HBD3_23–45_ (= peptide γ) and [S^33,40^]HBD3_23–41_. To verify this result, we prepared the latter peptide (peptide ε, Fig. 1A). When incubated in human serum, peptide ε was found intact after 24 h incubation (data not shown). We may conclude that the γ-core β-hairpin of HBD3 is highly resistant to hydrolysis by serum proteases, on par with full-length HBD3. Such pronounced serum stability may appear remarkable for a sequence devoid of a rigid structure and rich in basic amino acids, which are the preferred P1 residues of multiple serum proteases (trypsin, thrombin, plasmin, kallikrein etc)^49^; however, several studies have shown that cyclic peptides are not easily digested by serum proteases^49^,^50^, and indeed cyclization is an effective strategy to increase the serum stability of antimicrobial peptides^51^,^52^. It may be significant therefore that cyclization of the γ-core β-hairpin via disulfide bond formation is accelerated in serum versus aqueous buffer.

### The γ-core β-hairpin of HBD3 has antimicrobial activity comparable to the full-length peptide

**Figure 2:**
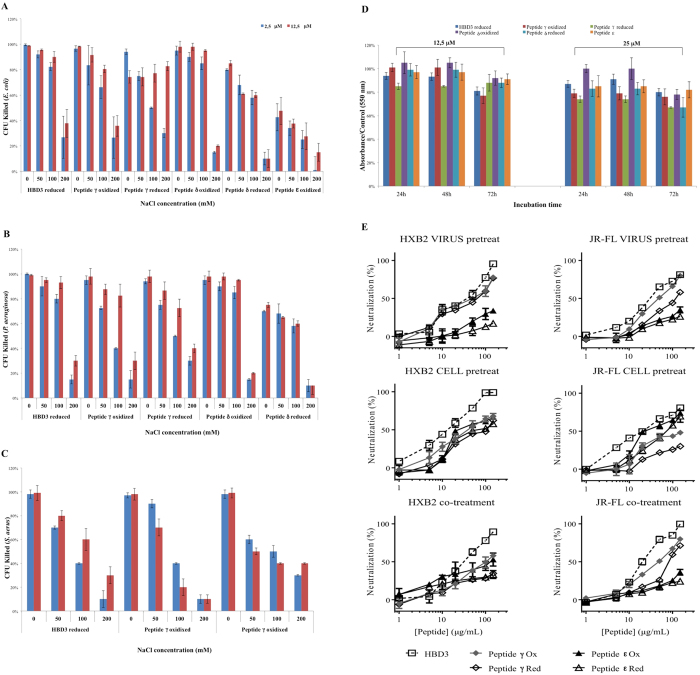
The γ-core peptides have potent antimicrobial activity without affecting cell viability. Antibacterial activity of peptides γ,δ,ε in comparison with full-length HBD3 on (**A**) *E. coli.* and (**B**) *P. aeruginosa*, and (**C**) *S. aureus* at the concentration of 2.5 μM (blue bars) and 12.5 μM (red bars), in the presence of the indicated concentrations of NaCl. Error bars show the standard deviations (SDs) from three independent experiments. Based on the absence of activity on *E. coli*, peptide δ was not tested on the other species. **(D)** Effects of the indicated peptides tested at the indicated concentration on A549 cells viability by MTT test. The data are expressed as the means ± standard error of three independent experiments. **(E)** Effects of the γ-core peptides on HIV infectivity. The γ-core peptides γ and ε were tested in oxidized and reduced form on the indicated HIV-1 viral strains at the concentration of 1, 5, 10, 20, 50, 100 and 150 μM, in comparison with reduced HBD3. Data expressed as % neutralization are from triplicates, with indicated SD. The same data in Table format are in the S.I. See also Tables S6, S7.

The antibacterial activity of peptides γ,δ,ε was evaluated in comparison with full-length HBD3. Having previously shown that the reduced and fully oxidized forms of HBD3 have comparable antibacterial activity^19^,^20^, we used reduced HBD3 as comparator (Fig. 2A–C). Two concentrations of each peptide (2.5 and 12.5 μM), were tested against the gram negative bacteria *E. coli* and *P. aeruginosa,* and the gram positive *S. aureus*, in the presence of increasing concentrations of NaCl (0, 50, 100 and 200 mM). In line with the HBD3 findings, the reduced and oxidized forms of peptides γ and ε had identical activity. At low ionic strength (without NaCl) both γ-core peptides displayed antimicrobial activity comparable to HBD3, but the activity was more salt-sensitive, being significantly lower at 50 and 100 mM salt. Notably, neither activity nor salt sensitivity differed significantly between peptide γ and ε, despite the considerable difference in their net positive charge. This contrasts with the hypothesis that the reduced salt sensitivity of HBD3 is simply a result of the very high positive charge of the molecule, and suggests that additional structural features, like increased affinity for the membrane or the ability to dimerize^41^, may play a role. In contrast to the γ-core peptides γ and ε, peptide δ (tested in the oxidized form) did not display significant antibacterial activity against *E. coli*., with <50% bacterial killing even in the absence of NaCl (Fig. 2A). This is a further demonstration that the β-hairpin closed by the Cys^18^-Cys^33^ disulfide may not represent the archetypal core of HBD3.

### The γ-core β-hairpin of HBD3 is not toxic to human cells

To evaluate if the antibacterial activity of the γ-core peptides is associated with cytotoxicity to human cells, we performed MTT test on A549 cells, with exposure times of 24, 48, and 72 h, using the γ-core peptides γ,δ, ε and HBD3 as comparator. Figure 2D shows the percent viability of cells exposed to the peptides at concentrations of 12.5 and 25.0 μM: at both concentrations, we observed only a slight reduction of cell viability (≤15% at 24 h, ≤25% at 48 h and 72 h) which was equivalent for all the peptides, including HBD3. We may conclude that the γ-core peptides are not cytotoxic for human cells.

### The γ-core β-hairpin of HBD3 binds CD98

We previously reported that HBD3 interacts with CD98, a type II transmembrane protein whose overexpression has been associated with increased attachment of *Enteropathogenic E. coli* and *C. rodentium* in mouse colon^31^, endocytosis of vaccinia virus^53^, and poor prognosis in several tumours^30^,^32^,^33^, and proposed that this interaction plays an important role in the antibacterial and innate immune surveillance activity of the peptide^21^. Using surface plasmon resonance (SPR) experiments we previously mapped the HBD3 binding site to a negatively charged cavity located between residues Val^304^-His^414^ of CD98^16^. Here we used SPR experiments to establish the affinity for CD98 of peptides γ and ε (Table 1). Peptide γ binds CD98 with 8-fold lower affinity than HBD3, while peptide ε, lacking the charged C-terminal region R^42^-K^45^, binds CD98 with 70-fold lower affinity. Importantly, both peptides bind CD98 in the same region previously identified for the full-length β-defensin (AA 304–414), which coincides with the region bound by proteins from *enteropathogenic E. coli* and *Citrobacter rodentium*^54^,^55^. These data are consistent with an interaction driven by electrostatic complementarity between the negatively charged residues lining the cavity at the top of CD98 and the two positive charge patches of HBD3^21^, which are both present in peptide γ, but only one of which is retained in peptide ε.

### The γ-core β-hairpin of HBD3 has antiviral activity

The antiviral activity of the γ-core peptides γ and ε in oxidized and reduced form was evaluated, in comparison with reduced HBD3, against HIV and HSV. To establish in which phase, if any, the peptides inhibit virus infectivity, we performed experiments in four conditions: a) peptides incubated with the virus before being added to the target cells (“virus pre-treatment”), b) peptides incubated with the cells before exposure to the virus (“cell pre-treatment”), c) simultaneous addition of peptides and virus to the target cells (“co-treatment”), and d) peptides added to the virus-exposed cells (“post-treatment”).

#### Antiviral activity against HIV

In all conditions and for both strains HXB2 and JR-FL, the behavior of the γ-core peptides paralleled that of HBD3, but with slightly reduced antiviral potency (Fig. 2E and Tables S6, S7). All the peptides were more efficacious when added to the cells (cell pre-treatment) or to the virus (virus pre-treatment) prior to addition of the virus to the target cells, then when added to the virus at the moment of the addition to the target cells (co-treatment). No activity was observed when the peptides were added after the virus (post-treatment). In pre-treatment conditions HBD3 and peptide γ showed 50% inhibition at 20 and 50 μM, respectively, for HXB2 and JF-RL; the reduced peptide was considerably less efficacious (50% inhibition at 100 and 150 μM). Peptide ε, lacking the charged R^42^-K^45^ region, was only active in cell-pre-treatment conditions, where it showed comparable potency to peptide ε.

#### Antiviral activity against HSV

As seen for HIV, the γ-core peptides showed similar, but slightly inferior, activity to HBD3 in all four conditions (Table 2). In agreement with prior reports^19^,^22^, HBD3 was more efficacious in cell pre-treatment and co-exposure experiments (50% inhibition at 10 μM) than in virus pre-exposure experiments (50% inhibition at 20 μM), while no efficacy was observed in post-exposure conditions. Oxidized peptide γ showed 50% inhibition at 100 μM in cell pre-treatment and co-exposure experiments, while it reached 50% inhibition at ≥150 μM in virus co-exposure experiments; the peptide was inactive in post-treatment conditions. Oxidized peptide ε was slightly less efficacious, and both peptides were less effective in reduced form. Overall, these results suggest that the antiviral activity of the γ-core peptides is exerted, like full-length HBD3, at the level of viral attachment and entry. Although they are relatively poor inhibitors, their IC_50_ is not far from the average concentration of β-defensins in mucosal epithelium (10–100 μg/mL)^22^, which is likely higher locally^10^, and would be increased upon virus exposure. Also of interest and unlike that seen for antibacterial activity, the presence of the disulfide improves the antiviral activity. This is in line with the different SAR requirements for the two activities^56^.

## Discussion

We asked whether the γ-core motif within HBDs represents a primordial HDP that evolved into the full-length molecules of today. To answer the question, we analyzed the structural and biological properties of the β-hairpin peptide of HBD3, which is the smallest substructure encompassing the γ-core motif (Fig. 1B). We first tried to establish if the γ-core β-hairpin represents the folding nucleus of the full-length HBD3. The results of our study of the oxidative folding of HBD3 were consistent with this hypothesis, but did not prove it since formation of the γ-core β-hairpin, closed by the Cys^23^-Cys^41^ disulfide bridge, occurred simultaneously with formation of the β-hairpin closed by the Cys^18^-Cys^33^ disulfide bridge. However, in the peptides corresponding to the two β-hairpins (peptides γ and δ in Fig. 1A) only the γ-core β-hairpin formed rapidly in neutral buffer, and even more rapidly in human serum. Based on these data, it is feasible that the γ-core β-hairpin is the folding nucleus of the β-sheet and subsequently, of the whole HBD3 molecule. Once oxidized, the γ-core β-hairpin is remarkably stable in human serum, despite the presence of trypsin-sensitive sites and the absence of a rigid 3D structure as assessed by nuclear magnetic resonance. These features are consistent with those of an HDP that is exported from a reducing cytoplasmic environment to exert antibacterial activity at the cell surface. Although stable in plasma, HBD3 is rapidly degraded by the elastolytic protease cathepsins, which are upregulated in the bronchoalveolar lavage of patients with emphysema or cystic fibrosis^57^. Taggart *et al.* reported that the primary inactivating cleavage sites of HBD3 by cathepsins reside in the N-terminal region and, notably, further trimming leads to a stable fragment, i.e., the sequence from 22 to 45 amino acids residues of HBD3, which is the hairpin of the full-length HBD3^57^.

We next evaluated whether the biological activities exerted by HBD3 were also exerted by the γ-core. We found that peptide γ displayed potent antibacterial activity against gram-negative (*E. coli* and *P. aeruginosa*) and gram-positive (*S. aureus*) bacteria (Fig. 2A–C). The absence of the R^41^-K^45^ C-terminal tail in peptide ε reduced, but did not eliminate these activities. Peptide γ also displayed the same antiviral activity as the full-length HBD3 against HIV (Fig. 2E) and HSV (Table 2). This antiviral activity was exerted at the same stage (entry) of the viral life cycle, and could be physiologically relevant in the case of HIV. Notably, the need for the disulfide bridge in the γ-core β-hairpin, which is important for the antiviral but not for the antibacterial activity, applies also to HBD3. Importantly, peptide γ displayed micromolar affinity for CD98, about 8-fold lower than the full-length HBD3, at the same binding site (Table 1). Binding to and downregulation of CD98 mediates the anti-invasive activity of HBD3 against pathogenic enteric bacteria^21^, and is likely to be important also for antiviral activity^28^,^29^,^30^,^31^,^32^. Overall, these results show that the γ-core β-hairpin of HBD3 is a viable HDP *per se*, and shares the broad antibacterial and antiviral activity of the full-length β-defensin.

The smallest HBD3 region thus far reported to maintain antimicrobial activity is the C-terminal decapeptide with the Cys residues mutated to Ser^58^. Krishnakumari *et al.*^59^,^60^ designed the single-disulfide analogue Phd3 corresponding to the C-terminal segment of HBD3, which is very similar to the sequence of our peptide γ but with one extra N-terminal residue; however, the two cysteine residues within the sequence, Cys^33^ and Cys^41^ (HBD3 numbering) are deleted, which might impede preservation of the native structure. We recently designed a small chimeric peptide designated “AMC”, constituted by the N-terminal region of HBD1 and the C-terminal region of HBD3, that exerts potent antibacterial activity in the presence of high concentrations of salt^61^,^62^.

Notably, the closest analog of peptide γ is C25P/L25P, which exerts antimicrobial activity^49^, and corresponds to the sequence of peptide γ with two extra N-terminal residues, and the internal Cys^33^ and Cys^40^ mutated to Trp and Ser, respectively. While C25P/L25P displays antimicrobial activity, the shorter peptide C21P/L21P, which largely overlaps our peptide δ, does not^37^. Also in agreement with our data, C25/L25P does not have a hemolytic effect on erythrocytes. Notably, L25P adopts a α-helical structure in the presence of anionic but not zwitterionic vescicles, which probably reflects its ability to disrupt bacterial, but not eukaryotic membranes^37^,^63^,^64^.

In summary, most of the previously reported HBD3 analogues have a sequence and a structure close to that of the γ-core and exert antimicrobial activity. This suggests that certain amino acids are highly conserved, while others have considerable plasticity^6^. Indeed, the loop of the γ-core hairpin (Gly^31^–Lys^39^ in HBD3), which is the longest loop region of all HBDs, contains sites of positive selection, consistent with its key functional role^65^.

**Figure 3:**
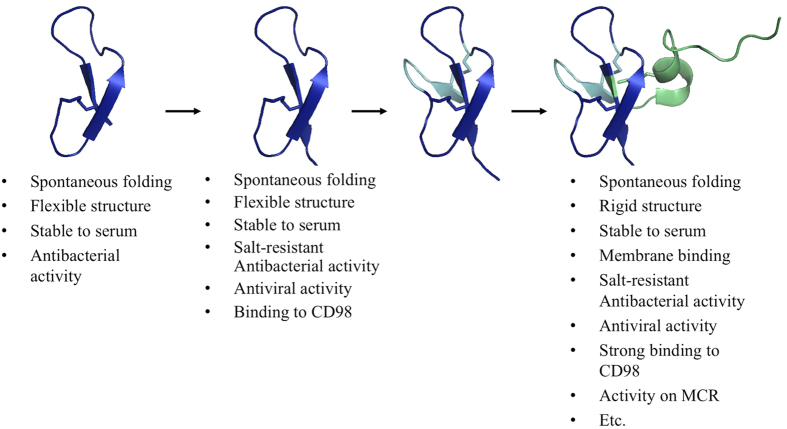
Hypothetical evolutionary pathway of HBD3. The structures along the pathway are shown, together with a schematic list of associated structural and biological features taken from the literature or established in the present work. For an extended description see text.

Yeoman & Yount identified the γ-core signature as an evolutionary starting point for a wide variety of cysteine-stabilized host defence molecules^2^,^9^,^10^,^11^, with a modular structure in which distinct domains execute specific functions. Within this paradigm, we may hypothesize an HBD3 evolutionary pathway (Fig. 3) that (i) starts with the ancestral single-disulfide γ-core with basal antimicrobial and antiviral activity; (ii) introduces the conserved Gly-X-Cys^4^ motif (Gly^31^-Lys^32^-Cys^33^ in HBD3), common to all β-Defensins^65^, which forms a β-bulge that induces a twist in the β-sheet structure^66^, enabling the addition of a third β-strand to form a β-sheet; (iii) adds the α-helical domain, which confers increased binding to the membrane^65^, increased activity against Gram-positive bacteria^58^, stronger salt resistance, activity on the melanocortin receptor^34^, chemotactic activity^65^,^67^ and other known or yet unexplored properties.

In a broader perspective, the identification of a disulfide-linked loop HDP (group 4 of Hancock and Sahl^4^) *within* a β-sheet HDP stabilized by multiple disulfides (group 2 of Hancock and Sahl^4^), further highlights the evolutionary relatedness of HDPs and blurs the boundaries between them.

Finally, besides providing insight into the evolution of β-defensins and HDPs based on the γ-core, our results might have some practical applications. In fact, a small peptide that recapitulates most of the biological properties of natural β-defensins could be used as a flexible base for the design of disease-specific compounds, with activity against antibiotic-resistant bacteria.

## Methods

### Peptide Synthesis and Purification

The peptides were synthesized on solid-phase using 9-fluorenylmethoxycarbonyl (Fmoc) chemistry protocols, as previously described^14^,^15^. Purification and LC/MS analysis were performed using a combined HPLC-ESI-MS system, and the identity of purified peptides was confirmed by ESI LC-MS. Disulfide bond formation was carried out by air oxidation at 1 mg/mL in 0.1M ammonium bicarbonate. Full details are given in the SI.

### Oxidative folding of HBD3 and γ-core peptides

Reduced HBD3 was dissolved in Tris HCl 100 mM pH 7.5 (final peptide concentration 0.5 mg/mL) and shaken in an open vessel, with periodic withdrawal of aliquots. After alkylation of the free thiol groups with iodoacetamide solution, 100 μg of each the aliquot were analysed through RP-HPLC-ESI-MS to evaluate the oxidation status. The remaining 200 μg were purified via RP-HPLC to eliminate excess iodoacetamide, lyophilized, and subjected to tryptic digestion. Tryptic fragments were then indentified through RP-HPLC-ESI-MS. The same procedure, except tryptic digestion, was used for peptides γ and ε. Full details are given in the SI.

### Serum Stability

The serum stability of peptides γ, δ and ε was assessed as previously described^34^. Full details are given in the SI.

### NMR Spectroscopy

NMR spectroscopy was carried out as previously described^68^. Full details are given in the SI.

### Antibacterial Activity

The antibacterial activity against *Escherichia coli* ATCC 25922, *Pseudomonas aeruginosa* ATCC 27853, and *Staphylococcus aureus* ATCC 6538P, was assessed as previously described^19^,^20^. Each assay was performed twice in triplicate. Bactericidal activity (mean ± SD) is expressed as the ratio between the number of colonies in the presence of peptide and the number of colonies on a control plate. See also SI.

### Cell culture and Cytotoxicity studies

Cytotoxicity of the peptides was assessed by the 3-[4.5-dimethylthiazol-2-yl]-2.5-dipheniltetrazolium bromide (MTT) assay as previously described^20^. For details, see SI.

### Antiviral Activity

The antiviral activity of the peptides against human immunodeficiency virus (HIV) and herpes simplex virus (HSV) was measured as previously described^19^,^20^,^69^. For details, see SI.

### Surface Plasmon Resonance

Surface Plasmon Resonance experiments were performed as previously described^21^. For details, see SI.

## Additional Information

**How to cite this article**: Nigro, E. *et al.* An ancestral host defence peptide within human β-defensin 3 recapitulates the antibacterial and antiviral activity of the full-length molecule. *Sci. Rep.* **5**, 18450; doi: 10.1038/srep18450 (2015).

## Supplementary Material

Supplementary Information

## Figures and Tables

**Table 1:** Kinetic and equilibrium constants for the interaction of CD98 with HBD3 and the γ-core peptides γ and ε.

Immobilized	In solution	*K*_*on1*_(×10^3^ M^−1^s^−1^)	*K*_*off1*_ (×10^−2^ s^−1^)	*K*_*on2*_ (x10^−3^ s^−1^)	*K*_*off2*_ (×10^−3^ s^−1^)	*K*_*D*_ (×10^−7^ M)
CD98_103-630_	HBD3	8.51 ± 0.05	2.10 ± 0.03	9.03 ± 0.06	1.930 ± 0.03	4.35 ± 0.06
CD98_103-630_	Peptide γ	3.91 ± 0.01	3.57 ± 0.07	8.29 ± 0.07	0.540 ± 0.06	0.55 ± 0.07
CD98_103-630_	Peptide ε	0.73 ± 0.02	5.48 ± 0.03	1.24 ± 0.04	0.128 ± 0.04	0.06 ± 0.04
CD98_304-414_	HBD3	4.65 ± 0.07	1.26 ± 0.03	5.44 ± 0.06	0.907 ± 0.007	3.86 ± 0.07
CD98_304-414_	Peptide γ	3.95 ± 0.04	3.44 ± 0.06	7.55 ± 0.06	0.152 ± 0.05	0.52 ± 0.06
CD98_304-414_	Peptide ε	0.71 ± 0.04	5.38 ± 0.05	5.67 ± 0.05	0.116 ± 0.06	0.06 ± 0.06

**Table 2:** Reduction in HSV Infectivity (% of control) upon treatment with γ-core peptides γ and ε or HBD3 at the indicated concentrations and conditions.

Peptide	Concentration (μM)							Condition
	1	5	10	20	50	100	150	
HBD3	15	20	36	55	67	79	94	Virus pre-treatment
HBD3	32	45	64	82	95	100	100	Cell pre-treatment
HBD3	18	30	50	80	97	100	100	Co-treatment
HBD3	0	0	0	0	5	7	12	Post-treatment
Peptide γ ox	0	6	15	20	28	38	47	Virus pre-treatment
Peptide γ red	0	0	8	18	20	31	36	Virus pre-treatment
Peptide γ ox	0	15	26	30	38	54	65	Cell pre-treatment
Peptide γ red	0	12	12	18	32	51	53	Cell pre-treatment
Peptide γ ox	15	20	23	31	46	53	68	Co-treatment
Peptide γ red	7	12	20	28	38	46	59	Co-treatment
Peptide γ ox	0	0	0	0	0	0	0	Post-treatment
Peptide γ red	0	0	0	0	0	0	0	Post-treatment
Peptide ε ox	0	0	7	12	19	28	33	Virus pre-treatment
Peptide ε red	0	0	3	8	16	26	31	Virus pre-treatment
Peptide ε ox	0	0	15	18	23	32	41	Cell pre-treatment
Peptide ε red	0	10	10	12	17	28	42	Cell pre-treatment
Peptide ε ox	12	18	21	36	38	42	57	Co-treatment
Peptide ε red	3	13	18	22	33	38	48	Co-treatment
Peptide ε ox	0	0	0	0	0	0	0	Post-treatment
Peptide ε red	0	0	0	0	0	0	0	Post-treatment
