# Supplementary material for: An ancestral host defence peptide within human β-defensin 3 recapitulates the antibacterial and antiviral activity of the full-length molecule
Source: Sci Rep. 2015 Dec 21;5:18450. doi: 10.1038/srep18450 (PMC4685272; doi:10.1038/srep18450)
Supplement: Supplementary Information [file srep18450-s1.pdf]

Supplemental Data

An ancestral host defence peptide within human β-defensin 3 recapitulates the antibacterial and antiviral activity of the full-length molecule

Ersilia Nigro, Irene Colavita, Daniela Sarnataro, Olga Scudiero, Gerardo Zambrano, Vincenzo Granata, Aurora Daniele, Alfonso Carotenuto, Veronica Folliero, Massimiliano Galdiero, Richard A. Urbanowicz, Jonathan K. Ball, Francesco Salvatore, Antonello Pessi.

Supplemental Experimental Procedures

Peptide Synthesis and Purification. All the Fmoc amino acids and the reagents Diisopropylethylamine (DIPEA), piperidine, Acetic anhydride, trifluoroacetic acid (TFA), triisopropylsilane (TIS), Ethanedithiol (EDT) Dichloromethane (DCM), *N*,*N*-dimethylformamide (DMF), tert-butyl methyl ether, N,N'-dicycloexylcarbodiimide (DCC), HPLC grade water and acetonitrile were obtained from Sigma Aldrich (St. Louis, MO). 1-Hydroxybenzotriazole hydrate (HOBT) and O-(Benzotriazol-1-yl)-*N*,*N*,*N*',*N*'-tetramethyluronium hexafluorophosphate (HBTU) were obtained from AnaSpec, Inc. (Fremont, CA). Rink-amide-MBHA resin (substitution 0.38 mmol/g) was obtained from Novabiochem (San Diego, CA). The peptides were synthesized by 9-fluorenylmethoxycarbonyl (Fmoc) chemistry protocols on a Biotage Syro Wave instrument at 50 µmol scale. Fmoc deprotection was with with 30% piperidine in DMF for 3 + 12 min at RT. Solutions of Fmoc-AA, HOBT/HBTU, and DIPEA were prepared at the concentration of 0.5M, 0.45M, and 1M in DMF, respectively. Coupling was with 5 fold excess of Fmoc-AA-OH with 1:1:2 AA/HOBT-HBTU/DIPEA for 40 min at RT. Double coupling was used for the hindered Fmoc-Arg(Pbf)-OH. At the end of the synthesis, the peptides were acetylated with two treatments (20 min each) with a 10% solution of acetic anhydride and 6% DIPEA in DMF. Cleavage was with 92:2.5:1:2.5:2 TFA/H_2O/TIS/EDT/Thioanisole for 4 hours at 25°C, followed by precipitated and washings in tert-butyl methyl ether. Peptide purification and LC/MS analysis were performed using a combined HPLC-ESI-MS system [HPLC (Gilson)/Flexar SQ 300 ESI-MS (Perkin Elmer)]. Analytical RP-HPLC made use of a Jupiter Proteo 10µm C18 250x4.6 mm column (Phenomenex), monitoring at 220 and 280 nm, flow rate 1.3 mL/min, and the solvent system H_2O/0.1% TFA (A) and CH_3CN/0.1% TFA (B), with a linear gradient 5–60% B over 30 min. Peptide were purified by semi-preparative RP-HPLC on a Jupiter Proteo 90 Å C18 column (10x250 mm, 10 µm) (Phenomenex), using a linear gradient 5–60% B over 25 min, with flow rate 10 mL/min. The

identity of purified peptides was confirmed by ESI LC-MS. MS scan was performed in the mass range 200–3000 m/z. Disulfide bond formation was carried out by stirring a solution of the purified peptide at 1mg/mL in 0.1M ammonium bicarbonate for 24 hours in an open vessel. Completion of the reaction was confirmed by LC/MS.

Oxidative folding of HBD3. Reduced HBD3 was dissolved in Tris HCl 100 mM pH 7.5 (final peptide concentration 0.5 mg/mL). Oxidative folding was achieved by shaking the peptide solution in an open vessel. Aliquots of the mixture (300μg) were withdrawn at different times (0, 0.5, 1.5, 3 h), and free thiol groups were alkylated by addition of 150 μL of a 10 mg/mL iodoacetamide solution, for 5 min in the dark. 100 μg of each sample were then analysed through RP-HPLC-ESI-MS to evaluate the oxidation status. The remaining 200 μg were purified via RP-HPLC to eliminate excess iodoacetamide. After lyophilization and before tryptic digestion, samples were dissolved in 55 mM ammonium bicarbonate (final peptide concentration 1mg/mL) at pH 8. Tryptic hydrolysis was carried out using an enzyme/substrate ratio of 1:100 (w/w), overnight at 37°C. Tryptic fragments were then indentified through RP-HPLC-ESI-MS.

Oxidative folding of Peptides γ and δ. The reduced peptides were dissolved in Tris HCl 100 mM pH 7.5 (final peptide concentration 0.5 mg/mL), and the peptide solution was stirred in an open vessel. Aliquots of the mixture (250μg) were withdrawn at different times (0, 0.5, 1.5, 3 h), and free thiol groups were alkylated by addition of 125 μL of a 10 mg/mL iodoacetamide solution, for 5 min in the dark. Each sample was then analysed through RP-HPLC-ESI-MS.

Serum Stability. Peptides **γ**, **δ** and **ε** was assessed as previously described[1]. Briefly, the peptides (150 μg) were incubated with human serum from an healthy donor [240 μl 20% (v/v) in PBS] at 37ºC for 24 h. Aliquots of 30 μL were removed at different times (0, 0.5, 1, 2, 3, 6, 8, and 24 h), mixed with 70 μl of 0.5% TFA to precipitate the protein fraction, and centrifuged at 16200xg. The supernatants were injected on a combined LC-ESI-MS system made of a high-performance liquid chromatograph (Gilson, Middleton, USA) interfaced with a Flexar SQ 300 mass spectrometer with electrospray ionization (Perkin Elmer, CA, USA), equipped with a 5 μm, 300-Å, 4.6x250 mm

Jupiter C_{18} column (Phenomenex, Aschaffenburg, Germany), and operated with a linear 30 min gradient of 5 – 60% acetonitrile in 0.1% acqueous TFA. ESI+MS scan was performed in the mass range 200 –3000 m/z.

NMR Spectroscopy. The samples for NMR spectroscopy were prepared by dissolving the appropriate amount of peptide in 0.45 ml of $^{1}H_2O$ (pH 5.5), 0.05 ml of $^{2}H_2O$ (Aldrich, Milwaukee, USA) to obtain a concentration 1-2 mM of peptide. For the sample in micelle solution, dodecyl-phosphocholine (DPC)-d_{38} was also added to a concentration of 200 mM. NMR spectra were recorded at 25 °C on a Varian INOVA 700 MHz spectrometer equipped with a z-gradient 5 mm triple-resonance probe head. The spectra were calibrated relative to [(2,2,3,3-tetradeuterio-3-(trimethylsilanyl)]propionic acid (TSP = 0.00 ppm) (MSD Isotopes, Montreal, Canada). One-dimensional (1D) NMR spectra were recorded in the Fourier mode with quadrature detection. The water signal was suppressed by gradient echo[2]. 2D DQF-COSY[3,4], TOCSY[5], and NOESY[6] spectra were recorded in the phase-sensitive mode using the method from States[7]. Data block sizes were 2048 addresses in t_2 and 512 equidistant t_1 values. Before Fourier transformation, the time domain data matrices were multiplied by shifted $\sin^2$ functions in both dimensions. A mixing time of 70 ms was used for the TOCSY experiments. NOESY experiments were run with mixing times in the range of 150-300 ms. The qualitative and quantitative analyses of DQF-COSY, TOCSY, and NOESY spectra, were obtained using the interactive program package XEASY[8]. $^{3}J_{HN\text{-}H\alpha}$ coupling constants were obtained from 1D ^{1}H NMR and 2D DQF-COSY spectra.

Antibacterial Activity. A colony-forming unit (CFU) assay of the antibacterial activity of all peptides was performed against *Escherichia coli* ATCC (American Type Culture Collection, Manassas, VA, USA) 25922, *Pseudomonas aeruginosa* ATCC 27853, and *Staphylococcus aureus* ATCC 6538P. The strains were grown under aerobic conditions in tryptic soy broth (Difco Laboratories, Detroit, MI) at 37°C and were incubated for 2 h at 37°C using two concentrations of peptide (2.5 and 12.5 μM) and 4 concentrations of NaCl (0, 50, 100 and 200 mM), as previously described[9,10]. Each assay was performed twice in triplicate. Bactericidal activity (mean ± SD) is

expressed as the ratio between the number of colonies in the presence of peptide and the number of colonies on a control plate.

Cell culture and Cytotoxicity studies – MTT test. Parental A549 cells stored in liquid nitrogen were thawed by gentle agitation of their vials for 2 min in a water bath at 37°C. After thawing, the content of each vial was transferred to a 75 cm^2 surface area, tissue culture flask, diluted with 90% Dulbecco's Modified Eagle's Minimal Essential Medium (DMEM) (Sigma-Aldrich St. Luois. MO. USA) supplemented with 10% fetal bovine serum (FBS) (Lonza Basel, Switzerland) and 1% L-glutamine (Sigma-Aldrich St. Luois, MO, USA). Then the cells were incubated for 24 h at 37°C in a 5% CO2 to allow the cells to grow and form a monolayer in the flask. Cells grown to 80 – 95% confluency were washed with phosphate buffer saline (PBS), trypsinized with 3mL of trypsin-EDTA (ethylenediaminetetraacetic) solution (1X) (Sigma-Aldrich St. Luis. MO. USA), diluted, counted and seeded ($4x10^3$ cells/200 µl per well) into a 96-well microtiter tissue culture plates for 24h in triplicate. The reduction of the proliferation of the cells was evaluated by the 3-[4.5-dimethylthiazol-2-yl]-2.5-dipheniltetrazolium bromide (MTT) (Sigma-Aldrich St. Luis, MO, USA) assay which measures metabolic changes. On the next day, cells were incubated in the presence or absence of γ-core peptides according to different schemes: after 24, 48, and 72 hours of incubation at 37 °C, and at 12.5 and 25 µM. Adherent cells were stained with MTT dye solution i.e. 20 µL of 1:10 diluted MTT stock solution (5 mg/mL) and incubated for 4 hours. After the incubation, we verified the presence of the violet crystals that normally indicate the occurred metabolization of the MTT. Then the medium was removed (180 µL) and 180 µL of dimethyl sulfoxide (DMSO) (Sigma-Aldrich St. Luois, MO, USA) was added to dissolve the MTT crystals. The eluted specific stain was measured by a spectrophotometer (550 nm). The proliferation index of the untreated cells was compared to the negative control (cell + medium without peptides). The experiments were performed in duplicate.

Antiviral Activity against Human Immunodeficiency Virus (HIV). HeLa TZM-bl cells were

grown in DMEM supplemented with 10% fetal calf serum and 1% non-essential amino acids (NEAA) and seeded (2×10^5) in 96-well Optilux plates (BD Biosciences) 24h before use. HIV-1 pseudotype viruses were produced as previously described[11]. All experiments were performed in triplicate, and conducted in parallel with no-peptide controls. The inhibitory effects of the peptides on entry of the HIV-1 pseudotype viruses were assessed in four ways: (i) For “co-exposure” experiments, TZM-bl cells were incubated with increasing concentrations of the peptides (1, 5, 10, 20, 50, 100 and 150 μM) and with the pseudotype virus for 1 h at 37°C; (ii) For “virus pre-exposure” experiments, 100 μl pseudotype virus was incubated with increasing concentrations of the peptides for 1h at 37°C, then added to TZM-bl cells plated into a 96-well Optilux plate; iii) For “cell pre-exposure” experiments, TZM-bl cells were incubated with increasing concentrations of the peptides for 30 min at 4°C, followed by addition of 100 μl pseudotype virus for 1h at 37°C; and iv) For “post-exposure” experiments, TZM-bl cells were incubated with virus for 1h at 37°C followed by addition of the peptides. All conditions had an additional 200 μl of medium added after 4 h and incubated for 72 hr. Medium was then removed, cells were lysed with 50 μl of cell lysis buffer (Promega), and 50 μl of luciferase substrate (Promega) was added. Luminescence was measured by using a BMG Labtech FLUOstar plate reader.

Antiviral Activity against Herpes Simplex Virus (HSV). Vero cells were grown in Dulbecco’s modified Eagle’s medium (DMEM) supplemented with 10% fetal calf serum. HSV-1 carrying a lacZ gene driven by the cytomegalovirus (CMV) IE-1 promoter to express beta-galactosidase was propagated as described elsewhere[12]. All experiments were conducted in parallel with no-peptide controls. The effect of peptides on the inhibition of HSV infectivity on cell monolayers[13] was assessed as follows: (i) For “co-exposure” experiments, the cells were incubated with increasing concentrations of the peptides (1, 5, 10, 20, 50, 100 and 150 μM) and with the viral inoculum for 45 min at 37°C. Non-penetrated viruses were inactivated by citrate buffer at pH 3.0. Monolayers were fixed, stained with 5-bromo-4-chloro-3-indolyl-beta-D-galactopyranoside (X-gal) and plaque

numbers were scored. Experiments were performed in triplicate and the % inhibition was calculated vs. the no-peptide control; (ii) For "virus pre-exposure" experiments, approximately 2 x 10^4 PFU of HSV-1 were incubated with increasing concentrations of the peptides for 45 min at 37°C, then titrated on Vero cell monolayers; iii) For "cell pre-exposure" experiments, Vero cells were incubated with increasing concentrations of the peptides for 30 min at 4°C, and infected with serial dilutions of HSV-1 for 45 min at 37°C; and iv) For "post-exposure" experiments, Vero cell monolayers were incubated with virus for 45 minutes at 37°C and afterwards the peptides were added to the inoculum, followed by an additional incubation period of 30 minutes at 37°C.

Surface Plasmon resonance. Surface Plasmon resonance (SPR) experiments were performed using a Biacore T200 system (GE Healthcare, Uppsala, Sweden), at 25 °C. Binding assays were carried out between various CD98 sequences and both reduced and oxidized HBD3. The residue numbering used here refers to CD98 isoform-1. GST-CD98 (102-630) and the truncated GST-CD98 (304-414) (Novus Biologicals, Littleton, CO) were immobilized on a CM4 and/or a CM5 sensor chip (research grade) by the standard amine coupling procedure, using HBS-EP buffer (HEPES 10 mM, NaCl 150 mM, EDTA 3 mM, Surfactant P20 0.005%, pH 7.4), as running buffer. CD98 was immobilized through activation of the sensor chip with 60 μL of N-hydroxysuccinimide and N-ethyl-N-(dimethylaminopropyl)-carbodiimide at 10 μL min^{-1}, followed by a 30 μL injection of protein diluted in 10 mM sodium acetate buffer, pH 4.0. Unreacted activated groups were blocked by a 60 μL injection of 1 M ethanolamine at 10 μL min^{-1}. The final amount of immobilized CD98 was typically between ~ 1500 and 5000 RU, corresponding to a protein concentration of 15 ÷ 50 mg/mL on the surface layer. Subsequently, the peptides or full-length HBD3 were injected at various concentrations (100 nM–100 μM), using HBS-EP as running buffer. The binding assays were performed under previously optimized conditions[14], i.e. a contact time of 120 s, a dissociation time of 1000 s, and a flow rate of 30 μL min^{-1}.

The SPR sensorgrams were analyzed by curve-fitting using the Biacore T200 Evaluation software. Dose response curves were plotted using the SPR sensorgrams and the corresponding fit. Various

reaction models were used to perform a complete kinetic analysis of the sensorgrams, and the best fitting was considered in such a way that the χ^2 value, representing the statistical closeness of curve-fitting, became the lowest. The two-state reaction model (including a conformational change) was chosen, which is described as:

A + B ⇆ AB ⇆ AB*

where A is the immobilized CD98 form, B is HBD3, AB represents the protein–peptide complex and AB* represents the stable complex after the conformational change. The binding dissociation constant (K_D) was calculated from the equation:

$$K_D = \frac{k_{off1}}{k_{on1}} \times \frac{k_{off2}}{(k_{off2} + k_{on2})}$$

T= 0

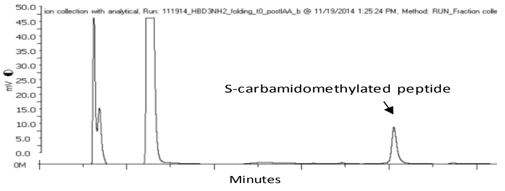

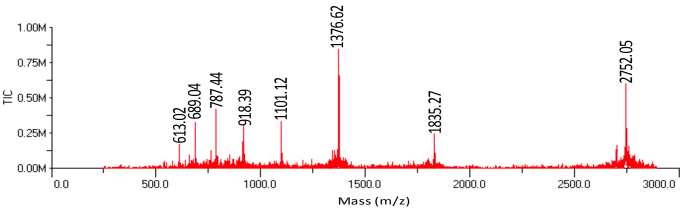

GIINTLQK / YY^{11}C^{CAM}R / VR / GGR / ^{18}C^{CAM}AVLS^{23}C^{CAM}LPK / EEQIGK / ^{33}C^{CAM}STR / GR / K / ^{40}C^{CAM}^{41}C^{CAM}R / RKK

RT	Peptide sequence	Experimental Mass (m/z)
5.33	GR	231.42
	GGR	288.61
7.6	C^{CAM}C^{CAM}R	494.62
	VR	273.52
14.7	EEQIGK	702.91
15.1	YYC^{CAM}R	660.51
18.9	GIINTLQK	886.21
19.1	C^{CAM}AVLSC^{CAM}LPK	1046.85
	C^{CAM}STR	524.01

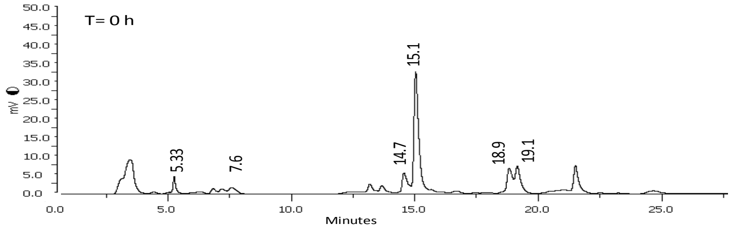

T= 0.5 h

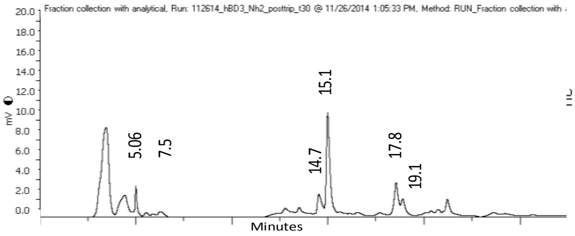

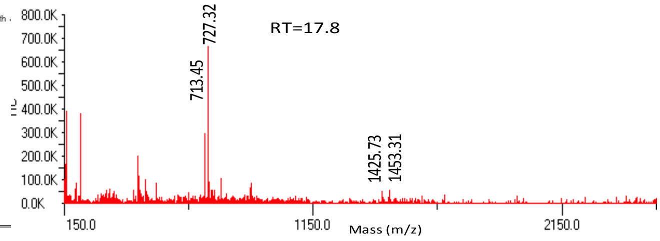

RT	Peptide sequence	Experimental Mass (m/z)
5.06	GR	231.58
	GGR	288.83
7.5	VR	273.62
14.7	EEQIGK	702.73
15.1	YYC(CAM)R	661.03
18.6	GIINTLQK	886.02
17.8	^{18}C^{CAM}AVLS^{23}CLPK ^{40}C^{CAM}^{41}CR (23-41 disulphide bond)	1425.73
	^{18}C^{CAM}AVLS^{23}CLPK ^{33}CSTR (18-33 disulphide bond)	1453.31
19.1	^{18}C^{CAM}AVLS^{23}C^{CAM}LPK	1047.06

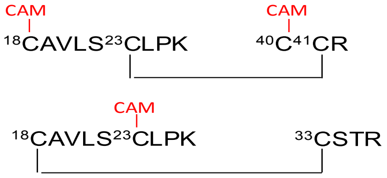

RT=17.8
MW= 1425.72

RT=17.8
MW= 1453.75

T= 1.5 h

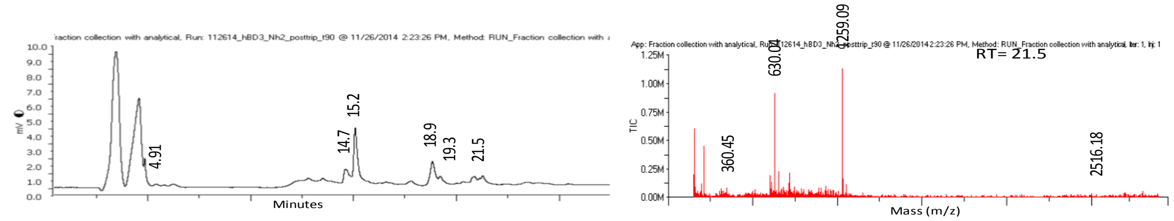

RT	Peptide sequence	Experimental Mass (m/z)
4.91	GR	231.59
	GGR	288.61
14.7	EEQIGK	703.03
15.2	YYC^{CAM}R	660.94
17.8	^{18}C^{CAM}AVLS^{23}CLPK ^{40}C^{CAM41}CR (23-41 disulphide bond)	1425.73
	^{18}C^{CAM}AVLS^{23}CLPK ^{33}CSTR (18-33 disulphide bond)	1453.31
18.9	GIINTLQK	886.22
19.3	^{18}C^{CAM}AVLS^{23}C^{CAM}LPK	1047.02
21.5	^{18}CAVLS^{23}CLPKEEQIGK ^{33}CSTR ^{40}C^{CAM41}CR (18-33, 23-41 disulphide bonds)	2516.18

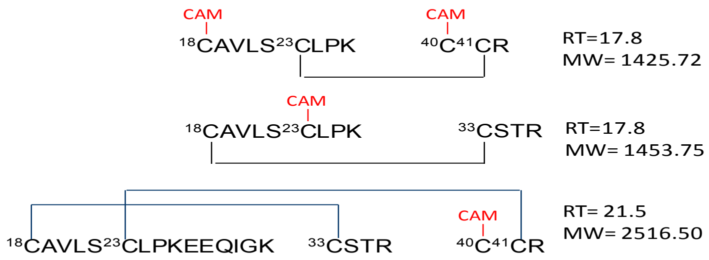

Figure S1. Time-course of the oxidative folding of 0.5 mg/mL HBD3 in Tris HCl, pH 7.5.

Aliquots of the folding mixture at various time points were treated with iodoacetamide, and excess reagent removed by HPLC. Following trypsin treatment, fragments were identified by MS. Shown is the HPLC trace after trypsin treatment, diagnostic MS spectra, and a list of the MS values at the indicated retention times. See also Table S1.

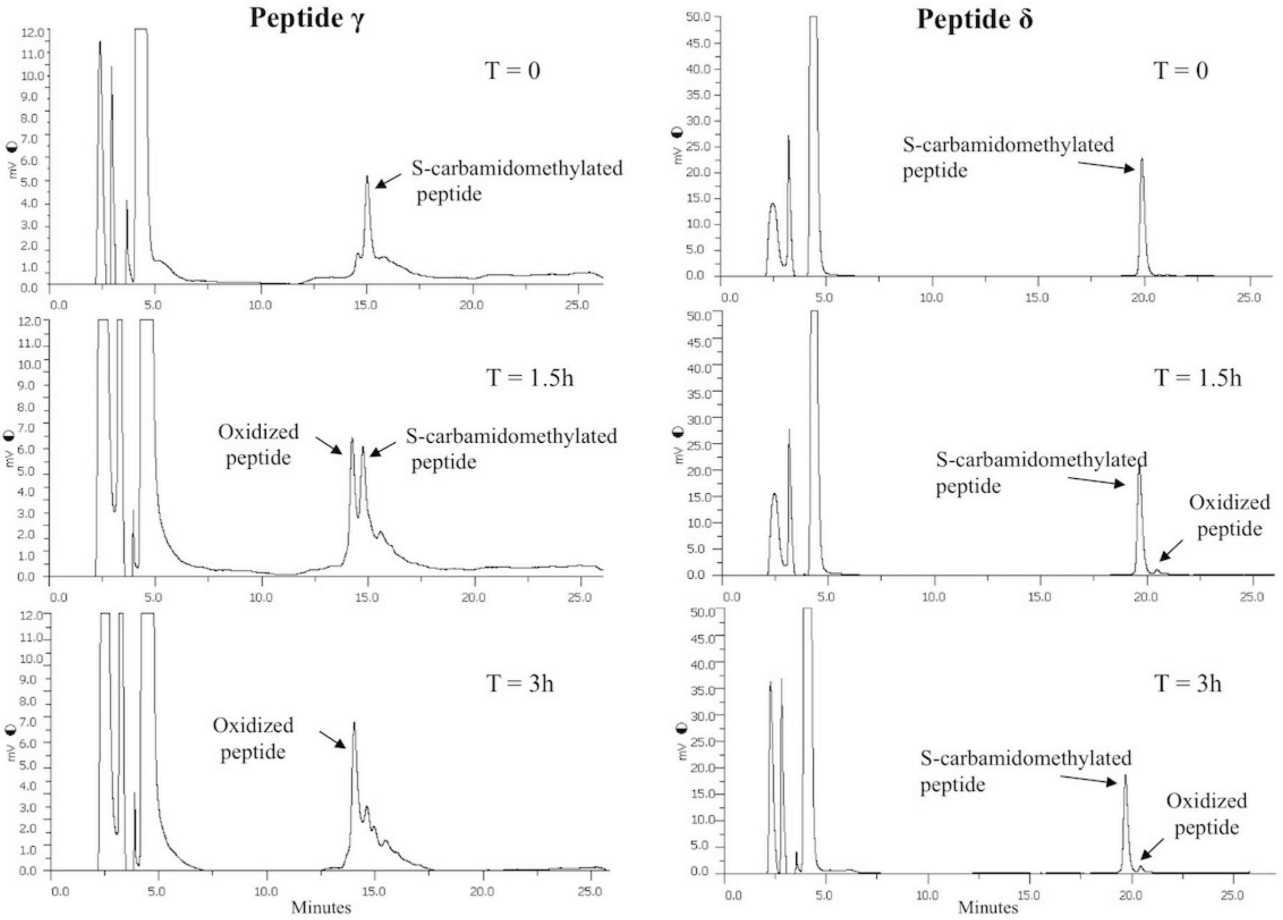

Figure S2. Time-course of the oxidation of 0.5 mg/mL peptides γ and δ (Figure 1A) in 100 mM Tris HCl pH 7.5. The identity of the peaks indicated as oxidized and reduced/carboxymethylated was established by MS.

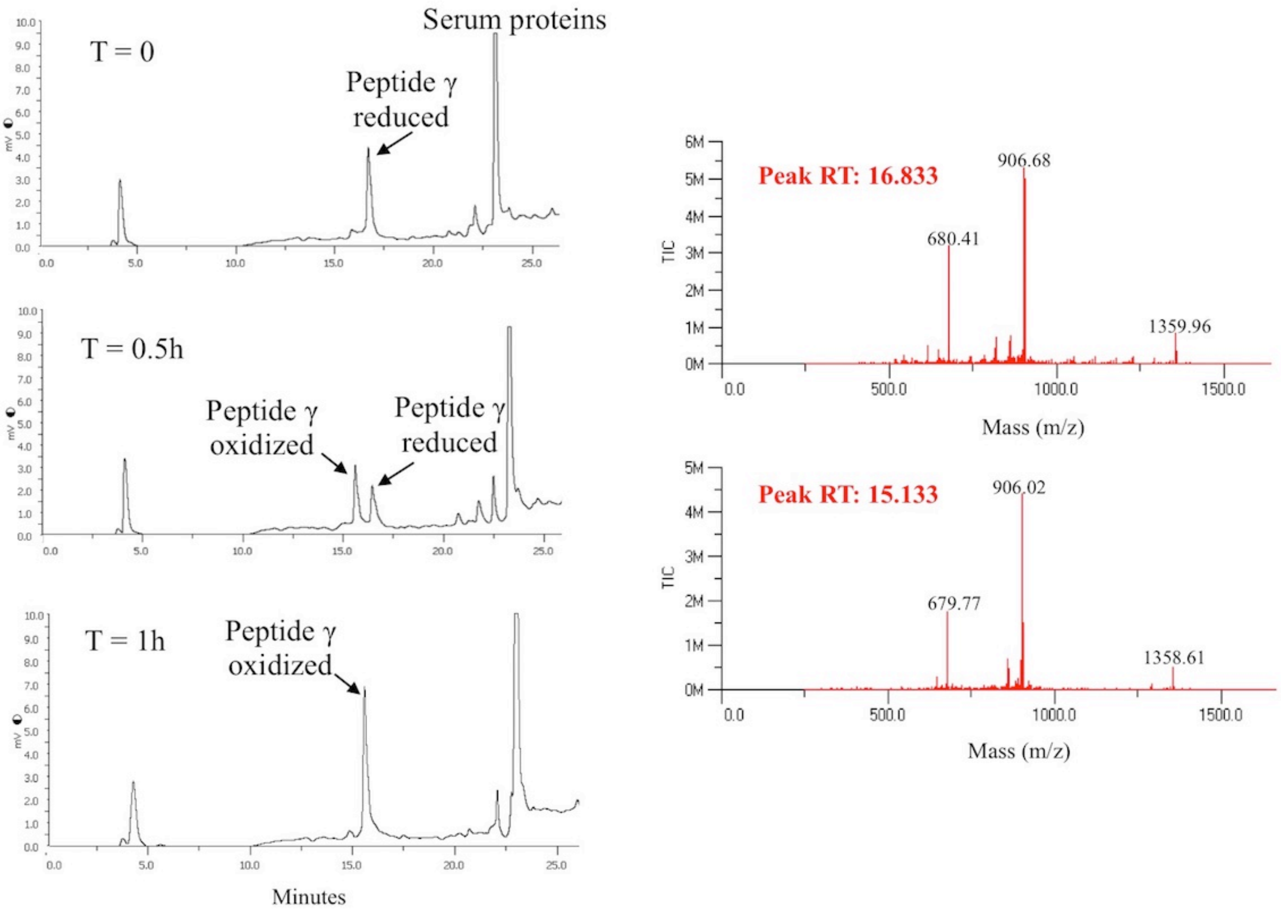

Figure S3. The γ-core β-hairpin disulfide Cys^{23}-Cys^{41} forms rapidly in human serum. (Left panels) Analytical RP-HPLC of the incubation of the peptide γ in human serum; (Right panels) ESI+full MS spectrum of the reduced (RT = 16.833 min) and oxidized (RT = 15.133 min) peptide. (Right panels) Multicharged ions at the corresponding peptide retention time. The theoretical mass of the peptide is 2717.24 Da (reduced form) and 2715.24 Da (oxidized form).

Table S1. Tryptic peptides identified at three time points during oxidative folding of HBD3. CAM, carbamidomethyl

		T = 0	T = 0.5 h	T = 1.5 h
**RT**	**M/z**	**Peptide Sequence**	**Peptide Sequence**	**Peptide Sequence**
5.3	231.42	GR	GR	GR
5.3	288.61	GGR	GGR	GGR
7.6	494.62	C^{40CAM}C^{41CAM}R		
7.6	273.52	VR	VR	VR
14.7	702.91	EEQIGK	EEQIGK	EEQIGK
15.1	660.51	YYC^{11CAM}R	YYC^{11CAM}R	YYC^{11CAM}R
17.8	1425.73		C^{18CAM}AVLSC^{23}LPK---C^{40CAM}C^{41}R<br>disulfide bond C^{23}-C^{41}	C^{18CAM}AVLSC^{23}LPK---C^{40CAM}C^{41}R<br>disulfide bond C^{23}-C^{41}
17.8	1453.31		C^{18}AVLSC^{23CAM}LPK---C^{33}STR<br>disulfide bond C^{18}-C^{33}	C^{18}AVLSC^{23CAM}LPK---C^{33}STR<br>disulfide bond C^{18}-C^{33}
18.9	886.21	GIINTLQK	GIINTLQK	GIINTLQK
19.1	1046.85	C^{18CAM}AVLSC^{23CAM}LPK	C^{18CAM}AVLSC^{23CAM}LPK	C^{18CAM}AVLSC^{23CAM}LPK
19.1	524.01	C^{33CAM}STR	C^{33CAM}STR	C^{33CAM}STR
21.5	2516.18			C^{18}AVLSC^{23}LPKEEQIGK---C^{33}STRC^{40CAM}C^{41}R<br>disulfide bonds C^{18}-C^{33} and C^{23}-C^{41}

Table S2. NMR Resonance Assignments[a] of peptide γ in H_2O

residue	NH ($^3J_{\alpha N}$, Hz)	C^{α}H (Δδ RC; Δδ Def)[b]	C^{β}H	Others
23 Cyss	8.43 (7.3)	4.72 (0.03; -0.06)	2.95, 3.22	
24 Leu	8.69 (7.2)	4.66 (0.05; 0.16)	1.57	1.70 (γ); 0.94 (δ)
25 Pro		4.40 (-0.03; 0.05)	1.94, 2.33	2.03, 2.09 (γ); 3.73, 3.89 (δ)
26 Lys	na[c]	4.20-4.36	~1.80	~1.46 (γ);~1.69 (δ); ~3.01 (ε)
27 Glu	8.37 (ov)[d]	4.30 (-0.02, -0.56)	1.99-2.10	2.27 (γ)
28 Glu	8.37 (ov)	4.35 (0.03;-0.41)	1.99-2.10	2.24 (γ)
29 Gln	8.36 (ov)	4.40 (0.06; -0.55)	2.01, 2.10	2.36 (γ)
30 Ile	8.20 (7.4)	4.17 (0.02;-0.55)	1.89	1.20 (γ); 0.91 (δ)
31 Gly	8.52 (5.9)	3.95, 4.04 (0.04; -0.30)		
32 Lys	8.24 (ov)	4.37 (0.05;-0.56)	~1.80	~1.46 (γ);~1.69 (δ); ~3.01 (ε)
33 Ser	8.38 (ov)	4.50 or 4.55	~3.90	
34 Ser	8.38 (ov)	4.50 or 4.55	~3.90	
35 Thr	8.23 (7.7)	4.37 (0.00; 0.10)	4.29	1.22 (γ)
36 Arg	na	~4.36	~1.80	~1.65 (γ);~3.22 (δ)
37 Gly	8.44 (6.1)	3.97 (0.01; 0.19/-0.14)		
38 Arg	8.18 (ov)	4.38 (0.04; -0.19)	~1.80	~1.65 (γ);~3.22 (δ)
39 Lys	na	4.20-4.36	~1.80	~1.46 (γ);~1.69 (δ); ~3.01 (ε)
40 Ser	8.38 (ov)	4.50 or 4.55	~3.90	
41 Cyss	na	4.78 (0.09, -0.69)		
42 Arg	na	~4.36	~1.80	~1.65 (γ);~3.22 (δ)
43 Arg	na	~4.36	~1.80	~1.65 (γ);~3.22 (δ)
44 Lys	na	4.20-4.36	~1.80	~1.46 (γ);~1.69 (δ); ~3.01 (ε)
45 Lys	na	4.20-4.36	~1.80	~1.46 (γ);~1.69 (δ); ~3.01 (ε)

[a]Obtained at pH = 5, with TSP (δ 0.00 ppm) as reference shift. Chemical shifts are accurate to ±0.02 ppm.

[b]Δδ RC = Chemical shift difference from random coil Hα values from Ref [29]. Δδ Def = Chemical shift difference from HBD3 proton resonances reported in Ref (Schibli, et al., 2002). [c]na: not assignable. [d]ov: overlapped.

Table S3. NMR Resonance Assignments[a] of peptide ε in H_2O

residue	NH (δ RC ; δ Def)	$C^{\alpha}H$ (Δδ RC ; Δδ Def)	$C^{\beta}H$ (δ RC ; δ Def)	Others
23 Cyss	8.44 (7.3)	4.67 or 4.70	2.95, 3.22	
24 Leu	8.70 (7.1)	4.70 (0.09, 0.12)	1.57	1.70 (γ); 0.94 (δ)
25 Pro		4.41 (-0.02, 0.04)	1.94; 2.33	2.03, 2.09 (γ); 3.73, 3.89 (δ)
26 Lys	na[c]	4.20-4.36	~1.80	~1.46 (γ);~1.69 (δ); ~3.01 (ε)
27 Glu	8.37 (ov)[d]	4.27 (-0.05, -0.59)	1.99-2.10	2.28 (γ)
28 Glu	8.37 (ov)	4.30 (-0.02, -0.46)		2.24 (γ)
29 Gln	8.36 (ov)	4.35 (0.01, -0.64)		2.37 (γ)
30 Ile	8.22 (7.5)	4.15 (0.00 ;-0.57)	1.89	1.20 (γ); 0.91 (δ)
31 Gly	8.51 (5.9)	3.95, 4.05 (0.04; -0.30)		
32 Lys	8.23 (ov)	4.36 (0.04;-0.57)	~1.80	~1.46 (γ);~1.69 (δ); ~3.01 (ε)
33 Ser	8.37 (ov)	4.51 or 4.55	~3.90	
34 Ser	8.37 (ov)	4.51 or 4.55	~3.90	
35 Thr	8.24 (7.7)	4.37 (0.00 ; 0.10)	4.33	1.23 (γ)
36 Arg	na	4.35	~1.80	~1.65 (γ);~3.22 (δ)
37 Gly	8.44 (6.0)	3.97 (0.01; 0.19/-0.14)		
38 Arg	8.18 (ov)	4.37 (0.03; -0.20)	~1.80	1.60-1.73 (γ); 3.16-3.24 (δ)
39 Lys	na	4.20-4.36	~1.80	~1.46 (γ);~1.69 (δ); ~3.01 (ε)
40 Ser	8.38 (ov)	4.51 or 4.55	~3.90	
41 Cyss	na	4.75 (0.06, -0.72)		

[a]Obtained at pH = 5, with TSP (δ 0.00 ppm) as reference shift. Chemical shifts are accurate to ±0.02 ppm.

[b]Δδ RC = Chemical shift difference from random coil Hα values from Ref [29]. Δδ Def = Chemical shift difference from HBD3 proton resonances reported in Ref (Schibli, et al., 2002). [c] na: not assignable. [d]ov: overlapped.

Table S4. NMR Resonance Assignments[a] of peptide **γ** in DPC

residue	NH ($^3J_{\alpha N}$, Hz)	$C^{\alpha}H$ (Δδ RC; Δδ Def)[b]	$C^{\beta}H$	Others
23 Cyss	8.39 (7.2)	4.66 (-0.03;- 0.12)	2.93, 3.16	
24 Leu	8.57 (7.0)	4.62 (0.01; 0.12)	1.55	1.64 (γ); 0.88 (δ)
25 Pro		4.37 (-0.06; 0.02)	1.90, 2.29	1.98, 2.04 (γ); 3.65, 3.83 (δ)
26 Lys	na	4.20-4.34	~1.80	1.40-1.46 (γ); 1.66 (δ);
27 Glu	8.44 (7.2)	4.24 (-0.08; -0.62)	2.00	2.23 (γ)
28 Glu	8.30 (ov)	4.29 (-0.03; -0.47)	2.02	2.22 (γ)
29 Gln	8.25 (ov)	4.33 (-0.01; -0.66)	1.96, 2.05	2.33 (γ); 6.86, 7.49 (ε)
30 Ile	8.12 (7.4)	4.11 (-0.04; -0.61)	1.84	1.46, 1.16, 0.88 (γ); 0.83 (δ)
31 Gly	8.49 (5.8)	3.90, 4.00 (-0.01; -0.16/-		
32 Lys	8.16 (7.6)	4.33 (0.01; -0.60)	1.74, 1.84	1.42 (γ); 1.63 (δ)
33 Ser	8.36 (7.3)	4.45 (-0.02; ---)	3.85	
34 Ser	8.38 (6.9)	4.51 (0.04; 0.15)	3.85	
35 Thr	8.20 (7.8)	4.33 (-0.04; 0.06)	4.25	1.18 (γ)
36 Arg	na[c]	4.28-4.33	1.74-1.80	1.62 (γ); 3.18 (δ)
37 Gly	8.40 (6.2)	3.93 (0.03; -0.17/0.15)		
38 Arg	8.16 (ov)	4.28-4.33	1.74-1.80	1.62 (γ); 3.18 (δ)
39 Lys	na	4.20-4.34	~1.80	1.40-1.46 (γ); 1.66 (δ);
40 Ser	8.30 (7.1)	4.46 (-0.01; ---)	3.83	
41 Cyss	8.43 (7.7)	4.74 (0.05; -0.73)	2.97, 3.18	
42 Arg	na	4.28-4.33	1.74-1.80	1.62 (γ); 3.18 (δ)
43 Arg	na	4.28-4.33	1.74-1.80	1.62 (γ); 3.18 (δ)
44 Lys	na	4.20-4.34	~1.80	1.40-1.46 (γ); 1.66 (δ);
45 Lys	na	4.20-4.34	~1.80	1.40-1.46 (γ); 1.66 (δ);

[a]Obtained at pH = 5, with TSP (δ 0.00 ppm) as reference shift. Chemical shifts are accurate to ±0.02 ppm. [b]Δδ RC = Chemical shift difference from random coil Hα values from Ref [29]. Δδ Def = Chemical shift difference from HBD3 proton resonances reported in Ref (Schibli, et al., 2002). [c]na: not assignable. [d]ov: overlapped.

Table S5. Time-course of the incubation of [$S^{33,40}$]$HBD3_{23\text{-}45}$ (peptide γ, Figure 1A) in human serum.

Time (h)	Peptide sequence	Mass (Theor.)	m/z (Exp.)	Charge
0	Ac-CLPKEEQIGKSSTRGRKSCRRKK-NH_2	2715.24	1357.79	+2
			906.05	+3
			680.10	+4
1	Ac-CLPKEEQIGKSSTRGRKSCRRKK-NH_2	2715.24	1358.58	+2
			906.02	+3
			679.81	+4
3	Ac-CLPKEEQIGKSSTRGRKSCRRKK-NH_2	2715.24	1358.36	+2
			906.13	+3
			679.81	+4
6	Ac-CLPKEEQIGKSSTRGRKSCRRKK-NH_2	2715.24	1358.58	+2
			906.02	+3
			679.71	+4
	Ac-CLPKEEQIGKSSTRGRKSCRRK-OH	2587.05	1294.95	+2
			863.21	+3
			647.31	+4
	Ac-CLPKEEQIGKSSTRGRKSCR-OH	2303.69	1152.77	+2
			768.92	+3
			576.84	+4
	Ac-CLPKEEQIGKSSTRGRKSC-OH	2147.5	1074.60	+2
			716.84	+3
			537.75	+4
8	Ac-CLPKEEQIGKSSTRGRKSCRRKK-NH_2	2715.24	1357.91	+2
			905.74	+3
			679.70	+4

	Ac-CLPKEEQIGKSSTRGRKSCRRK-OH	2587.05	1294.70	+2
			862.90	+3
			647.44	+4
	Ac-CLPKEEQIGKSSTRGRKSCR-OH	2303.69	1152.55	+2
			768.70	+3
			576.70	+4
	Ac-CLPKEEQIGKSSTRGRKSC-OH	2147.5	1074.13	+2
			716.65	+3
			537.47	+4
24	Ac-CLPKEEQIGKSSTRGRKSCRRKK-NH_2	2715.24	1358.17	+2
			905.96	+3
			679.68	+4
	Ac-CLPKEEQIGKSSTRGRKSC-OH	2147.5	1074.32	+2
			716.90	+3
			537.66	+4

Table S6. Reduction in HXB2 Infectivity (% of control) upon treatment with γ-core peptides γ and ε or HBD3 at the indicated concentrations and conditions

Peptide	Concentration (µM)							Condition
	**1**	**5**	**10**	**20**	**50**	**100**	**150**	
HBD3	4	6	35	40	55	78	96	Virus pre-treatment
HBD3	9	30	44	59	78	99	99	Cell pre-treatment
HBD3	2	4	23	38	63	78	89	Co-treatment
HBD3	0	0	0	0	0	0	0	Post-treatment
Peptide γ ox	0	11	35	40	50	60	78	Virus pre-treatment
Peptide γ red	0	12	30	36	45	59	77	Virus pre-treatment
Peptide γ ox	0	13	28	38	55	64	68	Cell pre-treatment
Peptide γ red	0	4	11	31	45	48	62	Cell pre-treatment
Peptide γ ox	0	9	13	21	39	49	58	Co-treatment
Peptide γ red	0	8	9	22	26	28	35	Co-treatment
Peptide γ ox	0	0	0	0	0	0	0	Post-treatment
Peptide γ red	0	0	0	0	0	0	0	Post-treatment
Peptide ε ox	0	0	1	6	20	30	34	Virus pre-treatment
Peptide ε red	0	0	0	2	7	13	17	Virus pre-treatment
Peptide ε ox	0	0	10	49	58	61	67	Cell pre-treatment
Peptide ε red	0	0	12	37	49	52	58	Cell pre-treatment
Peptide ε ox	7	20	31	34	40	45	53	Co-treatment
Peptide ε red	7	17	21	25	28	30	33	Co-treatment

Peptide ε ox	0	0	0	0	0	0	0	Post-treatment
Peptide ε red	0	0	0	0	0	0	0	Post-treatment

Table S7. Reduction in JR-FL Infectivity (% of control) upon treatment with γ-core peptides γ and ε or HBD3 at the indicated concentrations and conditions

**Peptide**	**Concentration (µM)**							**Condition**
	**1**	**5**	**10**	**20**	**50**	**100**	**150**	
HBD3	2	12	20	38	65	73	81	Virus pre-treatment
HBD3	0	28	41	49	66	71	81	Cell pre-treatment
HBD3	0	7	23	50	80	85	100	Co-treatment
HBD3	0	0	0	0	0	0	0	Post-treatment
Peptide γ ox	0	0	10	30	51	66	80	Virus pre-treatment
Peptide γ red	0	0	2	15	34	44	58	Virus pre-treatment
Peptide γ ox	0	0	7	31	43	44	48	Cell pre-treatment
Peptide γ red	0	0	0	13	22	26	30	Cell pre-treatment
Peptide γ ox	2	8	20	33	51	67	80	Co-treatment
Peptide γ red	0	2	9	17	26	60	71	Co-treatment
Peptide γ ox	0	0	0	0	0	0	0	Post-treatment
Peptide v red	0	0	0	0	0	0	0	Post-treatment
Peptide ε ox	0	0	4	14	22	27	35	Virus pre-treatment
Peptide ε red	0	0	0	10	18	23	26	Virus pre-treatment
Peptide ε ox	0	6	19	49	58	65	74	Cell pre-treatment
Peptide ε red	0	1	12	25	41	58	70	Cell pre-treatment
Peptide ε ox	0	7	10	12	18	25	36	Co-treatment
Peptide ε red	0	3	8	10	17	23	24	Co-treatment

Peptide ε ox	0	0	0	0	0	0	0	Post-treatment
Peptide ε red	0	0	0	0	0	0	0	Post-treatment

Supplemental References

1. Antcheva, N., Morgera, F., Creatti, L., Vaccari, L., Pag, U., Pacor, S., Shai, Y., et al. Artificial β-defensin based on a minimal defensin template. *Biochem. J.* **421,** 435-447 (2009).
2. Hwang, T.L., & Shaka, A.J. Water Suppression That Works. Excitation Sculpting Using Arbitrary Wave-Forms and Pulsed-Field Gradients. *J. Magn. Res.* **112,** 275-279 (1995).
3. Marion, D., & Wuthrich, K. Application of phase sensitive two-dimensional correlated spectroscopy (COSY) for measurements of 1H-1H spin-spin coupling constants in proteins. *Biochem. Biophys. Res. Commun.* **113,** 967-974 (1983).
4. Piantini, U., Sorensen, O.W., & Ernst, R.R. Multiple quantum filters for elucidating NMR coupling networks. *J. Am. Chem. Soc.* **104,** 6800-6801 (1982).
5. Braunschweiler, L., & Ernst, R.R. Coherence transfer by isotropic mixing: Application to proton correlation spectroscopy. *J. Magn. Res.* **53,** 521-528 (1983).
6. Jeener, J., Meier, B.H., Bachmann, P., & Ernst, R.R. Investigation of exchange processes by two-dimensional NMR spectroscopy. *J. Chem. Phys.* **71,** 4546-4553 (1979).
7. States, D.J., Haberkorn, R.A., & Ruben, D.J. A two-dimensional nuclear overhauser experiment with pure absorption phase in four quadrants. *J. Magn. Res.* **48,** 286-292 (1982).
8. Bartels, C., Xia, T.-h., Billeter, M., Güntert, P., & Wüthrich, K. The program XEASY for computer-supported NMR spectral analysis of biological macromolecules. *J. Biomol. NMR* **6,** 1-10 (1995).
9. Scudiero, O., Galdiero, S., Cantisani, M., Di Noto, R., Vitiello, M., Galdiero, M., et al. Novel synthetic, salt-Resistant analogs of human Beta-Defensins 1 and 3 endowed with enhanced antimicrobial activity. *Antimicrob. Ag. Chemother.* **54,** 2312-2322 (2010).
10. Scudiero, O., Galdiero, S., Nigro, E., Del Vecchio, L., Di Noto, R., Cantisani, M., et al. Chimeric Beta-Defensin Analogs, Including the Novel 3NI Analog, Display Salt-Resistant Antimicrobial Activity and Lack Toxicity in Human Epithelial Cell Lines. *Antimicrob. Ag. Chemother.* **57,** 1701-1708 (2013).
11. Lacek, K., Urbanowicz, R.A., Troise, F., De Lorenzo, C., Severino, V., Di Maro, A., et al. Dramatic Potentiation of the Antiviral Activity of HIV Antibodies by Cholesterol Conjugation. *J. Biol. Chem.* **289,** 35015-35028 (2014).
12. Galdiero, S., Falanga, A., Vitiello, M., D'Isanto, M., Cantisani, M., Kampanaraki, A., et al. Peptides containing membrane-interacting motifs inhibit herpes simplex virus type 1 infectivity. *Peptides* **29,** 1461-1471 (2008).
13. Tarallo, R., Carberry, T.P., Falanga, A., Vitiello, M., Galdiero, S., Galdiero, M., et al. Dendrimers functionalized with membrane-interacting peptides for viral inhibition. *Int. J. Nanomed.* **8,** 521-534 (2013).
14. Colavita, I., Nigro, E., Sarnataro, D., Scudiero, O., Granata, V., Daniele, A., et al. Membrane Protein 4F2/CD98 Is a Cell Surface Receptor Involved in the Internalization and Trafficking of Human β-Defensin 3 in Epithelial Cells. *Chem. Biol.* **22**, 217-228 (2015).
